# DynamicME: dynamic simulation and refinement of integrated models of metabolism and protein expression

**DOI:** 10.1186/s12918-018-0675-6

**Published:** 2019-01-09

**Authors:** Laurence Yang, Ali Ebrahim, Colton J. Lloyd, Michael A. Saunders, Bernhard O. Palsson

**Affiliations:** 1 Department of Bioengineering, University of California at San Diego, 9500 Gilman Drive, La Jolla, 92093 CA USA; 2 Department of Management Science and Engineering, Stanford University, 475 Via Ortega, Stanford, 94305 CA USA; 3 Novo Nordisk Foundation Center for Biosustainability, Technical University of Denmark, Kemitorvet 220, Kongens Lyngby, 2800 Denmark

**Keywords:** Constraint-based modeling, Metabolism, Proteome, Dynamic simulation, Batch culture

## Abstract

**Background:**

Genome-scale models of metabolism and macromolecular expression (ME models) enable systems-level computation of proteome allocation coupled to metabolic phenotype.

**Results:**

We develop DynamicME, an algorithm enabling time-course simulation of cell metabolism and protein expression. DynamicME correctly predicted the substrate utilization hierarchy on a mixed carbon substrate medium. We also found good agreement between predicted and measured time-course expression profiles. ME models involve considerably more parameters than metabolic models (M models). We thus generate an ensemble of models (each model having its rate constants perturbed), and then analyze the models by identifying archetypal time-course metabolite concentration profiles. Furthermore, we use a metaheuristic optimization method to calibrate ME model parameters using time-course measurements such as from a (fed-) batch culture. Finally, we show that constraints on protein concentration dynamics (“inertia”) alter the metabolic response to environmental fluctuations, including increased substrate-level phosphorylation and lowered oxidative phosphorylation.

**Conclusions:**

Overall, DynamicME provides a novel method for understanding proteome allocation and metabolism under complex and transient environments, and to utilize time-course cell culture data for model-based interpretation or model refinement.

**Electronic supplementary material:**

The online version of this article (10.1186/s12918-018-0675-6) contains supplementary material, which is available to authorized users.

## Background

Almost 70 years ago, Monod posited that the rate-limiting steps for exponential growth is expected to be distributed over hundreds or thousands of reactions that form an enzymatic reaction network. In the same study, he observed that *Escherichia coli* cultured in media consisting of two limiting carbon sources underwent two exponential growth phases separated by a short lag phase [1]—the phenomenon he coined diauxie. Today’s genome-scale models of *E. coli* now account for over 2,000 metabolic reactions, and over 4,000 steps involved in the macromolecular expression machinery [2–4]. Consequently, recent studies have been approaching the classic problem of understanding the mechanisms and constraints that govern cellular dynamics armed with a comprehensive view of the genome-scale enzymatic network.

### Genome-scale modeling of cell metabolism

Computing the genotype-phenotype relationship is a fundamental challenge for computational biologists. Constraint-based reconstruction and analysis (COBRA) provides one approach for systems-level computation of biological networks using genome-scale biochemical network reconstructions [5]. Flux Balance Analysis (FBA) [6] in particular simulates flux distributions through a metabolic network by optimizing a cellular objective, such as maximizing growth rate subject to physicochemical, regulatory and environmental constraints. COBRA has been used to address a large variety of biological problems [7], and many algorithmic extensions have been developed [8].

### Accounting for macromolecular constraints

In an important extension of FBA (FBAwMC), the hierarchy of substrate utilization in mixed carbon media was predicted correctly by imposing intracellular macromolecule crowding constraints [9]. The constraints imposed were based on approximate crowding coefficients for cytosolic enzymes based on estimated molar volume and catalytic efficiency.

Recently, genome-scale reconstructions have expanded significantly with development of integrated models of metabolism and macromolecular expression (ME models) [3, 4, 10–13]. ME models explicitly compute transcription and translation machinery requirements to support metabolic flux distributions. The latest *E. coli* ME models [11, 12] account for 80% of the proteome by mass and predict the allocation and limitation of proteome toward cellular functions during optimal growth [14]. Therefore, ME models considerably expand the scope of systems-level investigation and computation across multiple biological scales and processes.

### Dynamic simulation of cell metabolism and macromolecular composition

Constraint-based models of metabolism have been used in a dynamic simulation framework to investigate by-product secretion [15], diauxic growth [16], transcriptional regulation [17], and metabolic engineering strategies [18, 19]. Recent studies have also incorporated the dynamics of protein expression. For example, temporal resource allocation was studied using a model of the cyanobacterium *Synechocystis* sp. PCC 6803 [20]. The model consisted of 52 reactions and 50 compounds, and also included coarse-grained reactions for synthesis of macromolecules including ribosome and multiple enzymes. Meanwhile, Waldherr et al. [21] performed a detailed mathematical study on the problem of predicting the dynamics of protein expression and metabolism. They developed Dynamic enzyme-cost FBA (deFBA), which accounts for the dynamics of cell metabolism, biomass production, and biomass composition. The framework accounts also for enzymatic capacity and the cost of their production. The approach could predict dynamic adaptation of enzyme expression from an optimization principle. The method was demonstrated on a core carbon metabolism model of *E. coli*.

### Objectives and outline of this study

Here, we develop a method to simulate the cellular dynamics of metabolism, protein expression, and macromolecular composition in response to environmental changes. We demonstrate a mathematically simple approach with a focus on applying it to a large, comprehensive network. The largest network we simulate consists of 7,027 molecular components (small molecules and macromolecules) and 12,677 reactions involved in metabolic and protein expression processes [2].

**Fig. 1:**
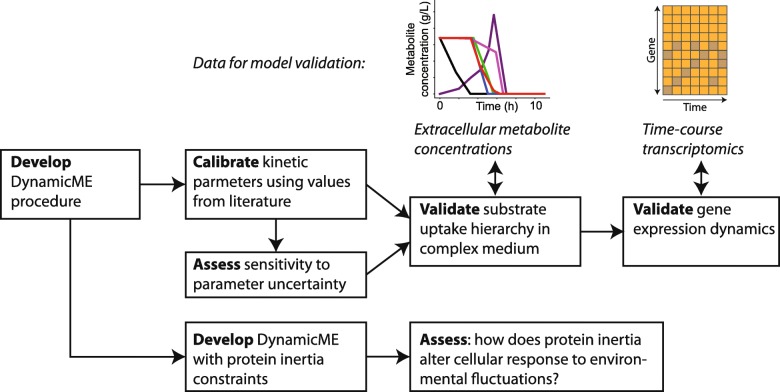
Schematic of the overall workflow for this study

The rest of this study is organized as follows. In Methods, we first briefly overview the relevant concepts for computing cell phenotype using a ME model. We then describe our main contribution, the DynamicME procedure. We first derive a simple approach for dynamic simulation using ME models, and extend this procedure to account for protein “inertia” constraints. We then describe the methods used for model parameter sensitivity analysis and model validation used in the rest of the paper. In Results, we apply DynamicME to the case study of batch growth of *E. coli* on a mixed carbon substrate medium. We then address the challenge of interpreting model simulations when many uncertain parameters are present by generating an ensemble of models with perturbed parameters. These models are analyzed with archetypal analysis to identify prominent time-course metabolite profiles. The overall workflow for this study is shown in Fig. 1.

## Methods

### Growth maximization for ME models

A ME model describes a cell’s metabolic and macromolecular state as a vector of *n* fluxes, $v\in \mathbb {R}^{n}$ (in mmol/grams dry weight/h) that catalyze biochemical reactions among *m* components (i.e., small molecules and macromolecules) [2]. To compute the state that maximizes the growth rate *μ* (in h ^−1^), one solves the following optimization problem (1) [2, 22]:

1 $$ \begin{aligned} \max_{\mu, v} \quad & \mu \\ \mathrm{subject\ to} \quad & S(\mu) v = 0 \\ & l(\mu) \leq v \leq u(\mu), \end{aligned}  $$

where $S(\mu)\in \mathbb {R}^{m\times n}$ is the stoichiometric matrix, and $l(\mu)\in \mathbb {R}^{n}$, $u(\mu) \in \mathbb {R}^{n}$ are the lower and upper flux bounds. These three parameters are functions of *μ*, for example, due to the hyperbolic relation between growth rate and translation rate, macromolecule dilution, etc. (see [2] for a complete description of these relations). Problem (1) includes constraints in the form of *S*(*μ*)*v*=0, where for any fixed *μ*, we obtain a linear program. Because our objective function here is to maximize *mu*, subject to the *μ*-dependent constraints (*S*(*μ*)*v*=0), a global optimum is found efficiently by bisecting on *μ*, or using augmented Lagrangian methods [22]. We note that similar optimization problems have also been solved in the context of metabolism and protein expression networks using the Resource Balance Analysis modeling framework (see SI Text E1 in [23] and [24]).

To solve (1), we used the solveME Python module [22]. Specifically, we used bisection (binary search) as in [11] to maximize growth rate to six decimal points. SolveME uses the 128-bit (quad-precision) linear program (LP) and nonlinear program (NLP) solver Quad MINOS 5.6 (qMINOS) [25, 26]. All qMINOS runs were performed with feasibility and optimality tolerances of 10^−20^. These tight tolerances were used to capture solutions involving fluxes as small as 10^−16^ mmol/gDW/h and were made possible through the quad-precision capabilities of qMINOS. The models used for this study are available in the Github repositories COBRAme (version 0.0.9) https://github.com/SBRG/ecoli_me_testing and ECOLIme (version 0.0.9) https://github.com/SBRG/ecolime. The COBRAme software [2] was used for building and developing the ME model.

### Dynamic simulation using ME models

Our DynamicME implementation extends dynamic FBA (dFBA) [15, 16], which was developed for metabolic models (M-models). We tested two implementations of the DynamicME method: one that does not account for proteome dynamic constraints (protein “inertia”) and one that does.

The first implementation assumes that the protein abundances can be adjusted freely between time steps. Also, the uptake rate of a substrate was not made a function of its extracellular concentration. Instead, flux bounds were set to zero if the substrate was depleted (zero concentration), or to a finite value otherwise. Consequently, we did not need to perform a ME-model simulation at every time step. Instead, once exchange (i.e., uptake and secretion) fluxes were computed by the ME-model, the same fluxes were used to compute the extracellular metabolite concentration profile over subsequent time steps. At each time step, DynamicME checked whether a substrate became depleted (fully consumed) or newly available, e.g., by feeding for a fed-batch process or secretion of re-consumable metabolites. If so, a new ME computation was performed with the updated exchange flux bounds. Here, the ME model is capable of selecting the optimal set of metabolites to take up from the medium. The exchange fluxes and growth rate were then updated according to the new optimal solution. These updated values were used to compute biomass and metabolite concentrations. This procedure was repeated until the batch time was reached.

**Fig. 2:**
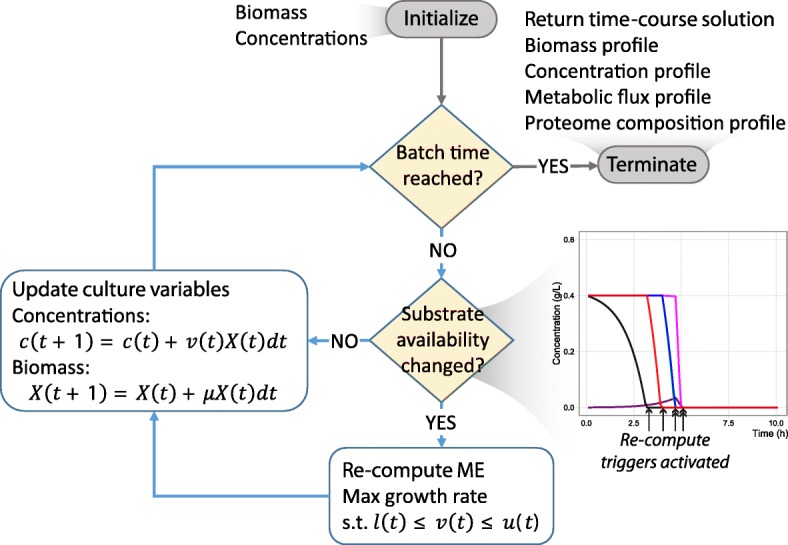
Schematic of the DynamicME procedure. The culture is divided into smaller time steps and extracellular concentrations and biomass are updated at each timepoint. Metabolite exchange fluxes are computed whenever substrate availability changes due to metabolite depletion, feed, or secretion. With each ME simulation, the metabolic flux distribution and proteome composition are also updated


In this first implementation, one can still account for concentration-dependent uptake rates or different feed schedules by performing ME-model simulations at every time step. Furthermore, if additional mechanisms such as growth inhibition by substrates or products are modeled, one should perform ME-model simulations at every time step. The procedure for simulating a batch culture using dynamicME is described in Procedure DynamicME. This implementation of DynamicME is also shown schematically in Fig. 2.

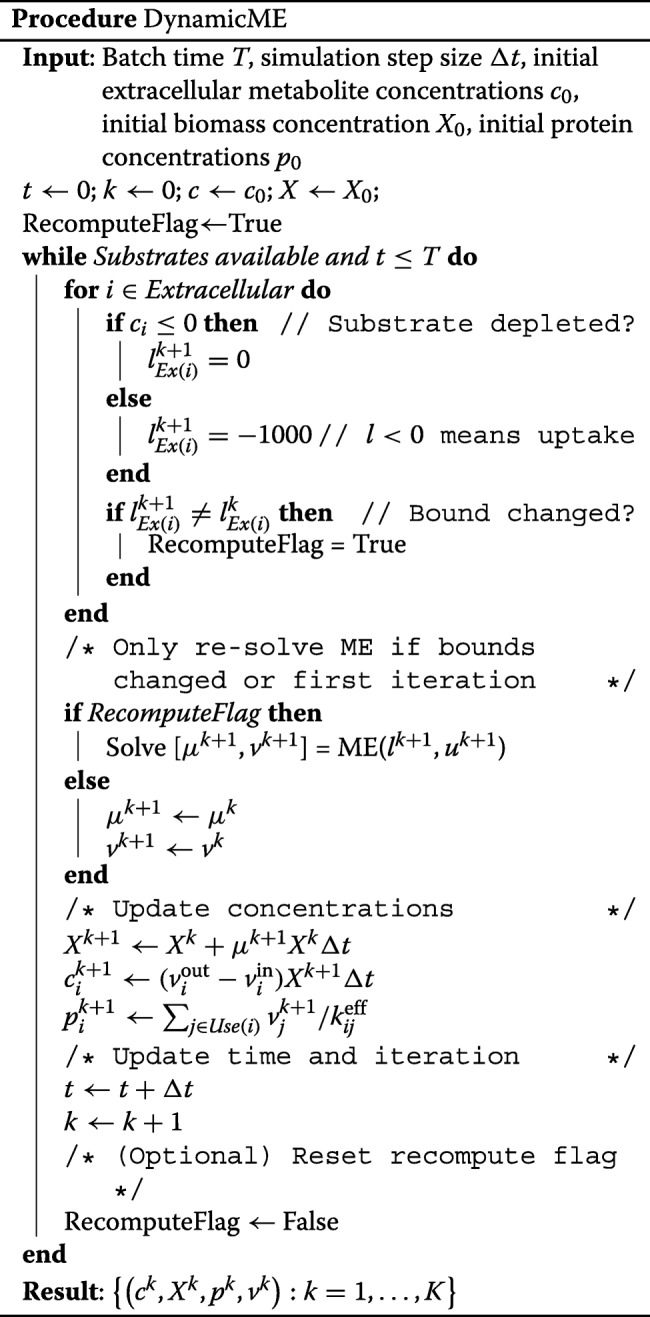


### DynamicME with protein inertia constraints

The second implementation accounts for protein abundance at the previous time step (i.e., protein “inertia”). This implementation requires modifying the ME model formulation. Thus, at each time step, we solve the following optimization problem (2):

2 $$ \begin{aligned} \max_{\mu, v,p, \delta} \quad & \mu \\ \mathrm{s.t.} \quad & S(\mu) v = 0 \\ & v^{\text{form}}_{i} - \mu p_{i} = \delta_{i}, \ \forall i \in {Complex} \\ & \sum_{j\in {CAT}(i)} \frac{v_{ij}}{k^{\text{eff}}_{ij}} \leq p_{i}, \ \forall i \in {Complex} \\ & p_{i} = p^{0}_{i} + \delta_{i} H \\ & l(\mu) \leq v \leq u(\mu) \\ & v_{i}^{\text{form}}\geq 0, \ \forall i \in {Complex} \\ & p_{i} \geq 0, \ \forall i \in {Complex}, \end{aligned}  $$

where *μ* is the growth rate, $v\in \mathbb {R}^{n}$ the vector of fluxes (metabolic and expression processes), $v_{i}^{\text {form}} \geq 0$ the flux of protein complex formation reaction for complex *i* (ComplexFormation reactions in the underlying ME model [2] that convert protein subunits to a complex according to defined complex stoichiometry), $p \geq 0 \in \mathbb {R}^{k}$ the vector of protein complex concentrations, $\delta \in \mathbb {R}^{k}$ the vector of protein complex concentration differences, $p^{0} \in \mathbb {R}^{k}$ the vector of protein complex concentrations at the previous time step, $S\in \mathbb {R}^{m\times n}$ the stoichiometric matrix that constrains the *m* components (metabolites and macromolecules), and *l*, *u* are the lower and upper flux bounds. *H* is the time horizon (in hours), which determines the anticipated time window in which to re-allocate the proteome. The value of *H* need not be the same as the simulation time step when (2) is solved inside the procedure InertiaDynamicME. In this study, we used *H*=2 hours. *Complex* is the set of protein complexes that are dynamically constrained, and *CAT* (*i*) is the set of reactions that are catalyzed by protein complex (enzyme) *i*. Note that the original ME network reconstruction accounts for mass balance of all metabolites and macromolecules, including protein complexes. Because we are now allowing accumulation (*δ*_*i*_>0) or depletion of complexes (*δ*_*i*_<0), we remove from *S*(*μ*)*v*=0 the mass balance constraints on protein complexes in the set *Complex*, and instead use the constraints $v_{i}^{\text {form}} - \mu p_{i} = \delta _{i}, \ \forall i \in {Complex}$.

In the protein inertia implementation, we solve Problem (2) at every iteration, rather than only re-solving when environmental conditions change. Re-solving at every iteration is necessary because the intracellular protein abundances are now potentially changing at every iteration, i.e., when *δ*_*i*_≠0; therefore, the metabolic fluxes are also subject to change at every iteration, whether or not extracellular conditions are changing. The inertia-constrained dynamicME procedure is described in InertiaDynamicME.


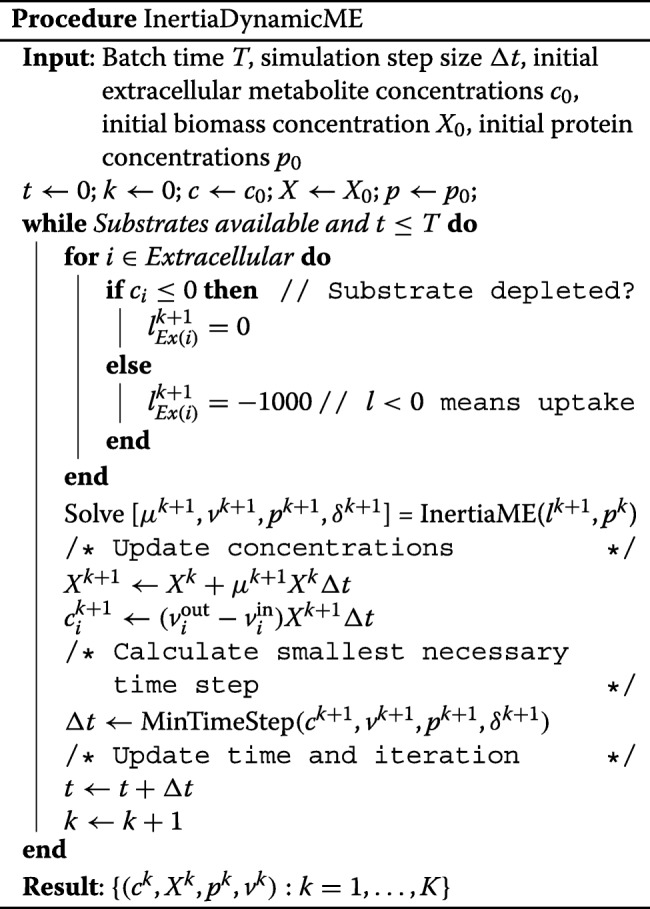


### Variable time step procedure (MinTimeStep)

At every time step, we compute the concentration for the next time step based on the current concentration *c*_*i*_ for each extracellular metabolite *i*. If the updated concentration would have become negative, i.e., the time step was too large, we then compute a new time step according to the formula: *Δt*^new,M^= min{*c*_*i*_/(−*v*_*i*_*X*):*i*=1,…,*p*}, where *p* is the number of extracellular metabolites whose concentrations are simulated, and *X* is the biomass concentration.

Similarly, we also compute a new time step if an intracellular protein concentration is detected to fall below zero with the current time step. In this case, we use the following formula: *Δt*^new,P^= min{*p*_*i*_/(*δ*_*i*_):*i*=1,…,*k*}. The time step is finally computed as *Δt*= min{*Δt*^0^,*Δt*^new,M^,*Δt*^new,P^}. If the time step *Δt* differs from *Δt*^0^, we re-solve the optimization problem using the updated time step. This way, we ensure that changes to the environment or intracellular concentrations are accounted for in the simulation, regardless of the initial time step.

### Model calibration using literature data

A number of adaptive laboratory evolution (ALE) studies have now demonstrated that the proteome of wild-type *E. coli* is not optimally allocated or efficient for every single nutrient [27–29]. To accurately reflect this wild-type proteome state, we calibrated the model with respect to several known enzymatic features of wild-type *E. coli*. First, glycerol kinase is known to be significantly less efficient for wild-type compared to ALE endpoints [30]. Second, ALE on lactate minimal medium showed multiple limitations in lactate utilization and enzymes near the phosphoenolpyruvate (PEP) node [27]. Third, respiration is known to have higher proteomic cost than fermentation, leading to acetate overflow [31]. Based on these observations, we calibrated the effective rate constants (*k*_eff_) (see section below for details). For example, we imposed a realistic turnover rate for isocitrate dehydrogenase based on literature data, effectively increasing proteomic cost for respiration. All calibrated parameters are listed in Additional file 1: Table S1 along with their original and adjusted values. Also, oxygen uptake rate was constrained to −20 mmol/gDW/h to reflect transport limitations not reflected in the proteome cost model.

### Sensitivity analysis of dynamic simulations

In the ME model, effective rate constants (*k*_eff_) relate metabolic flux *v* to enzyme concentration by the relationship *v*=*k*_eff_·*e*, where *e* is the enzyme concentration [11]. Precise estimates for these parameters are not available for many reactions and enzymes; therefore, an important step in ME model-based studies has been to assess sensitivity of predictions to these uncertainties [32]. In this study, we investigated the sensitivity of DynamicME predictions to uncertainties in *k*_eff_. We perturbed *k*_eff_ values from 0.1 to 10 times the nominal values. To avoid exploring the full parameter space consisting of thousands of *k*_eff_ values, we chose relevant pathways and perturbed only these reactions (Additional file 1: Table S1). We generated 200 random samples. Perturbed ME models having good fit to measured metabolite concentration profiles were treated as an ensemble. The exact determination of ensembles is described below.

### Archetypal analysis and ensemble of models

**Fig. 3:**
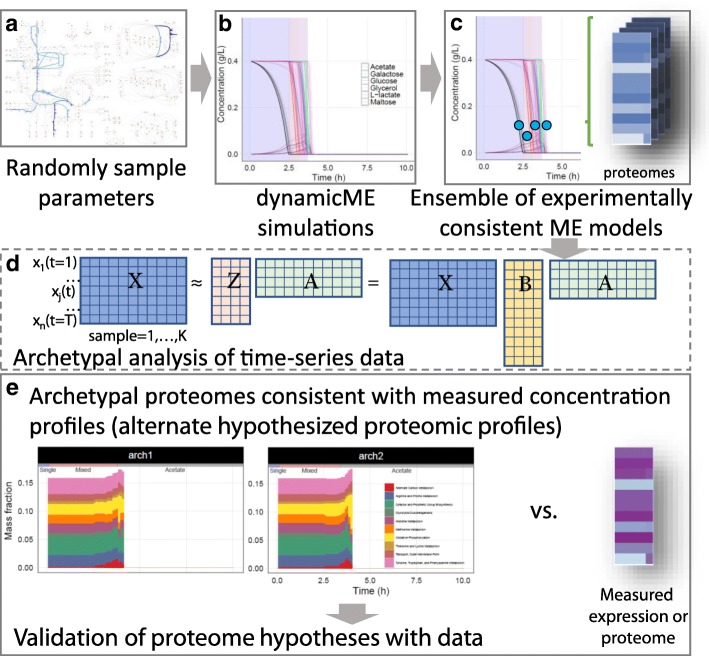
Ensemble model estimation and characterization procedure. (**a**-**c**) An ensemble of models with prediction error within a threshold was found by perturbing model parameters (*k*_eff_). **d** Archetypal analysis was used to characterize the ensemble where any data point is approximated as a convex combination of archetypes. **e** The predicted time-course proteome profile from the ensemble with best fit to measured metabolite concentrations was compared with measured time-course expression profiles

Archetypal analysis [33–35] is a dimension-reduction method in which any data point is approximated as a convex combination of the computed archetypes; in turn, each archetype is a convex combination of the data points [33]. Each archetype lies on the convex hull of the data and represents a “pure” phenotype. In our study, we performed archetypal analysis on randomly perturbed samples of model-predicted time-course metabolite concentration profiles (Fig. 3). Thus, each archetype represents a distinct phenotype with a particular substrate utilization hierarchy.

To prepare data for archetypal analysis, timepoints and metabolites were collapsed, resulting in a 2D matrix of features (timepoints-and-metabolites) × samples. In archetypal analysis, we then approximate *X* as *X*≈*ZA*, where *Z* is the matrix of archetypes and *A* is a matrix of coefficients with the constraints *A*_*ij*_≥0 and $\sum _{j=1}^{p} A_{ij}=1$ for *p* archetypes (Fig. 3d). Thus, *X* is approximated as a convex combination of archetypes. The matrix of archetypes *Z* is constrained as *Z*=*XB*, with the coefficient matrix *B*_*ij*_≥0, $\sum _{j=1}^{p} B_{ij}=1$ for *p* archetypes; therefore, the archetypes are constrained to be convex combinations of the data points *X*.

Once archetypes were determined, the proteome and exchange flux dynamic profiles were also mapped to the archetypes using *B*. The best number of archetypes was chosen using the elbow method from a scree plot [36] (Additional file 2: Figure S1). Archetypal analysis was performed using the spams Python module [35].

### Optimal parameter estimation via metaheuristic optimization

We developed an optimization-based procedure to match time-course concentration profiles by estimating *k*_eff_ values. For optimization, we used a gradient-free metaheuristic method (list-based threshold accepting) [37] because of its efficiency and flexibility. We developed a parallel implementation of this optimization method for increased efficiency (Additional file 3: Figure S2). The implementation allows each parallel node (CPU thread) to choose between following its local search trajectory or restarting the search from the current best solution. This parallel communication was implemented using MPI (Message Passing Interface) via the mpi4py Python module [38]. The objective function was the sum of squared errors between measured and predicted extracellular metabolite concentration profiles.

### Model validation using time-series expression profiles

To validate proteome allocation predictions, we computed the time-lagged cross-correlation between simulated and measured time-course proteome profiles. Lagged cross-correlation measures the similarity between two time-series where one lags the other, and has been particularly useful for analyzing time-course expression profiles. For example, it was used to study regulatory interactions of galactose metabolism in *E. coli* [39]. To compute lagged cross-correlation we used the R function ccf [40].

We obtained microarray hybridization intensity values over time points from Beg et al. [9]. We log2-transformed these values for further analysis. The log2-transformed measurements were compared against simulated protein mass fractions. We define mass fraction in two ways. First, for the ME model without protein inertia constraints, the protein mass fraction $f_{j} = v^{\text {trsl}}_{j} w_{j}/\sum _{j\in Prot}\left (v^{\text {trsl}} w_{j}\right)$, where $v_{j}^{\text {trsl}}$ is the translation rate of protein *j*, *w*_*j*_ is its molecular weight, and *Prot* is the set of all proteins in the ME model. Second, for the ME model with protein inertia constraints, the mass fraction $f_{j} = p_{j} w_{j} / \sum _{j\in {Prot}} (p_{j} w_{j})$, where *p*_*j*_ is the enzyme concentration, which is a variable in this modified ME model.

We first made the time intervals consistent between the measured and simulated expression profiles. To do so, we determined the smallest time interval used (i.e., measured or simulated) for the two profiles and linearly interpolated each profile separately using this time interval. In this study, the time interval was 0.1 h.

To determine the lagged cross-correlation for the entire simulated proteome, we iterated through each lag value, ranging from −1.7 to 1.7 h, and chose the lag corresponding to the highest median cross-correlation across all proteins.

## Results

### Growth on mixed substrates

When grown on complex media, *E. coli* uses substrates preferentially or simultaneously, depending on growth conditions [9]. Without additional constraints, FBA may erroneously predict simultaneous uptake of all substrates [9]. FBA with molecular crowding (FBAwMC) improves FBA by adding molecular crowding constraints, and correctly predicted substrate utilization hierarchy under a five-carbon medium [9].

We hypothesized that proteome-limited cellular growth would exhibit a hierarchy of preferential and simultaneous substrate utilization on mixed substrate media. To test this hypothesis, we implemented the DynamicME procedure: namely, time-course simulation of genome-scale integrated models of metabolism and macromolecular expression (ME-models) (Fig. 2). DynamicME extends dynamic FBA (dFBA) [16] to ME-models (see “Methods” section).

Using DynamicME, we simulated cellular growth on the five-carbon mixed substrate media studied by Beg et al. [9] and the simulated metabolite concentration profiles were compared with measurements. To simulate growth on nutrient-excess batch culture, carbon substrate uptake rates were effectively unconstrained (i.e., lower bound =−1000 mmol/gDW/h). Therefore, total proteome limitation became the active constraint rather than nutrient limitation.

**Fig. 4:**
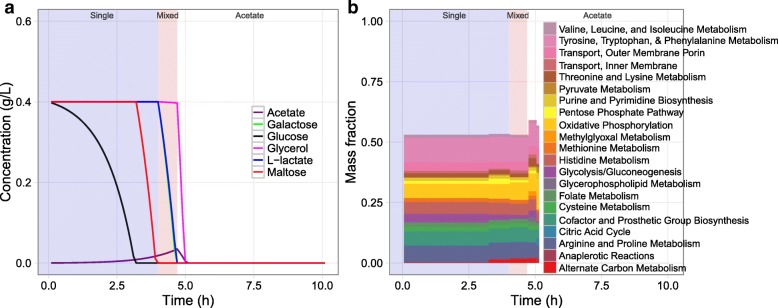
Predicted substrate uptake hierarchy and proteome allocation. **a** Predicted time-course metabolite concentration profile. **b** Predicted time-course proteome mass fraction allocation profile

DynamicME correctly predicted the majority of substrate uptake hierarchy characteristics, including the single substrate utilization (glucose), mixed utilization, and acetate reconsumption phases observed by Beg et al. [9] (Fig. 4). We found a few differences between simulated and measured profiles. Overall, metabolites were consumed more rapidly than experimentally observed. Also, acetate secretion was lower than measured, and maltose was predicted to be utilized earlier than in experiments.

In the absence of additional constraints, FBA was shown to predict optimal states that accurately reflect ALE (adaptive laboratory evolution) endpoints but may exceed the efficiency of wild-type cells [28, 41–43]. To account for this discrepancy, we implemented a model-calibration procedure to reflect observed metabolic and expression profiles better, as described in the following section.

### Model calibration for experimentally consistent concentration time-course profiles

Both the rate and hierarchy of substrate utilization are affected by ME-model parameters. In particular, the effective rate constants *k*_eff_ influence predicted pathway usage [44, 45]. We thus investigated the sensitivity of predicted substrate utilization hierarchy to uncertainty in *k*_eff_ values.

**Fig. 5:**
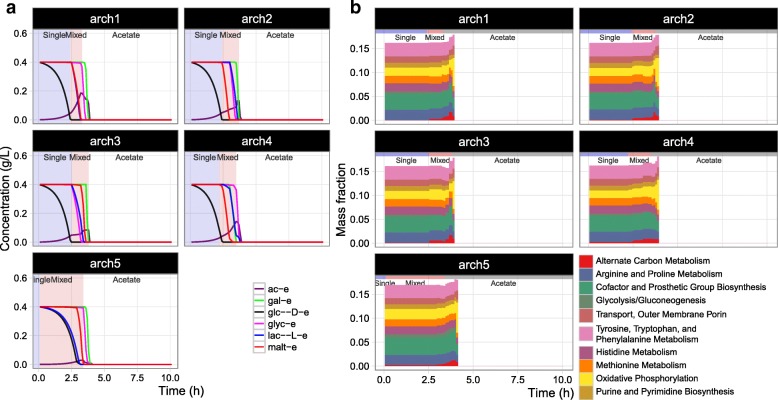
Sensitivity and archetypal analysis. Archetypal time-course concentration (**a**) and proteome mass fraction allocation (**b**) profiles were computed from simulations with 200 randomly perturbed *k*_eff_ parameters

First, we performed 200 random perturbations of *k*_eff_ values and performed DynamicME simulations for the perturbed models. The predictions showed large variations with respect to substrate utilization hierarchy. To aid interpretation, we performed archetypal analysis [33, 34] on the time-course metabolite concentration profiles and identified five archetypes (Fig. 5) as described in “Methods” section. The five archetypes showed considerable variation in substrate utilization hierarchy, reflecting the sensitivity of predictions to uncertainty in *k*_eff_ values. Of the five archetypes, archetype 4 most closely resembled experiments (Fig. 5). The archetypal model correctly predicted the sequence of glucose uptake followed by mixed utilization of maltose, lactate, and galactose, and finally glycerol uptake and acetate re-consumption. The acetate secretion rate was also significantly higher than the initial model and matched measurements better.

We also implemented an alternative approach to fit measured concentrations using metaheuristic optimization (Additional files 3: Figure S2 and 4: Figure S3). The optimal profiles were similar to that of archetype 4. Thus, we proceeded with subsequent analyses using archetype 4, which in turn represents an ensemble of experimentally-consistent ME models with differing parameter values.

### Predicting time-course proteome allocation

An important novelty of DynamicME is explicit computation of proteome allocation over a time-course simulation. For the mixed substrate medium, DynamicME computed distinct proteome compositions over time, corresponding to the changing metabolic modes (Fig. 4). We compared computed proteome dynamics with measured time-series microarray data [9]. For validation, we used the proteome profile from the most accurate archetype (archetype 4) as determined in the previous section (Fig. 5).

**Fig. 6:**
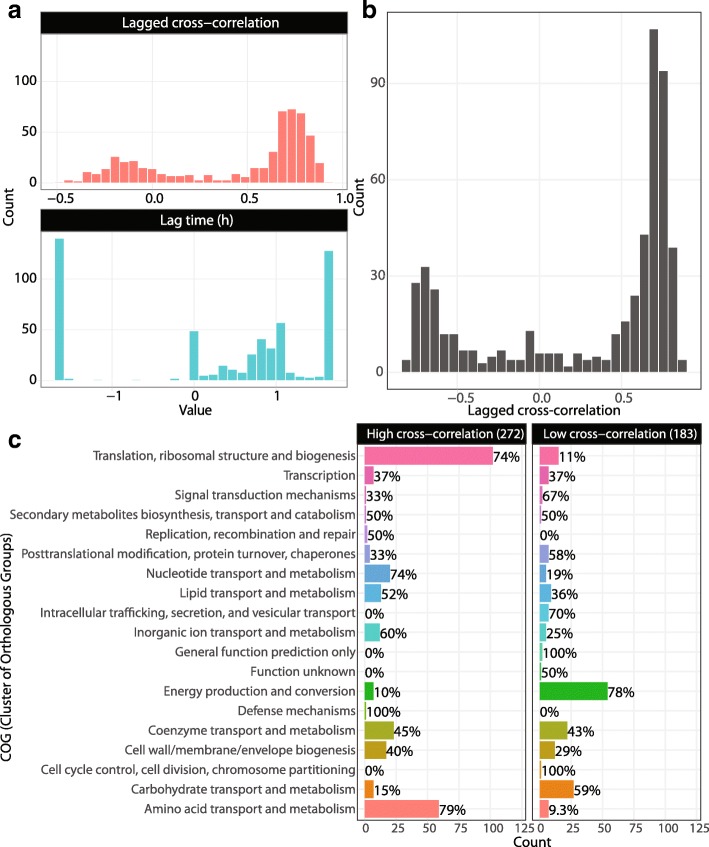
Lagged cross-correlation of simulated vs. measured expression. **a** Histograms of lagged cross-correlation values and lag time. **b** Histogram of cross-correlation values for fixed lag time of 1.2 h. **c** Functional groups (COGs) of genes with cross-correlations for fixed lag of 1.2 h, which were low (below 0.25) or high (above 0.64, the median)

To validate proteome allocation predictions, we computed the lagged cross-correlation [46] between simulated and measured time-course proteome profiles (Fig. 6). The lag time resulting in the highest median cross-correlation across all compared proteins was 1.2 h, indicating that proteome dynamics were faster than measured, which was consistent with metabolite concentration profiles. With this fixed lag time, the median lagged cross-correlation across proteins was 0.64 with values ranging from −0.83 to 0.86 (Fig. 6b).

In addition, certain functional gene sets were predicted better than others. For example, of 138 genes in the COG (Cluster of Orthologous Groups) [47] “Translation, ribosomal structure and biogenesis”, DynamicME predicted 74% with high (above 0.64, the median) and 11% with low (below 0.25) cross-correlation, respectively (Fig. 6c). Similarly, “nucleotide transport and metabolism,” “amino acid transport and metabolism,” and “inorganic ion transport and metabolism” were predicted with high cross-correlation. In contrast, of 69 “Energy production and conversion” genes, 78% had low cross-correlation. Closer inspection of these energy metabolism genes showed that the main discrepancy lay in genes related to oxidative phosphorylation: NADH dehydrogenase, cytochrome oxidase, ATP synthase, and citric acid cycle (Additional file 5: Table S2). The acetate secretion rate for the archetype 4 simulations were lower than measured (Fig. 5a), which was consistent with the discrepancy in gene expression dynamics. We next sought to investigate whether additional constraints could resolve some of these discrepancies.

### Effects of protein inertia on dynamic metabolic and protein expression profiles

Next, we investigated the effect of dynamic constraints on intracellular protein abundances. We assume that the protein concentration at a simulation time step depends on the concentration at the previous time step and the rates of synthesis, dilution, and degradation of the protein. Here, we do not account for active protein degradation. We assume that the synthesis rate is constrained by the transcription and translation capacity at that time step, which are computed based on the metabolic and expression network reconstruction and parameters of the underlying ME network used. Dilution rate is determined by growth rate and protein concentration. While not accounted for here, degradation rate depends on the capacity of the proteostasis machinery. Overall, the effect of additional constraints on protein dynamics on the optimal cellular response to environment change is not straightforward to deduce without a network-level model because they are determined by the metabolic and proteomic states of the cell, which change over time.

We hypothesized that the optimal cellular response to changing environments should differ between the scenarios of (a) instantaneous proteome reallocation versus (b) reallocation with dynamic constraints. This hypothesis has been investigated on a coarse-grained model by [48], who showed that proteome adaptation time is theoretically minimized by sequentially synthesizing the set of rate-limiting proteins via an on-off control strategy. Related to this hypothesis is the observation that *E. coli* expresses proteins that are not needed immediately [13, 32, 49]. This strategy of protein pre-allocation, which enables an increase in these proteins in less time, may provide fitness benefits when alternative carbon sources are encountered [32]. Additionally, when adaptation time is constrained, increased allocation of expression machinery is potentially advantageous to ensure rapid expression—e.g., by allocating a ribosome reserve under feast-famine cycles [13].

To test our hypothesis, we extended DynamicME and implemented proteome dynamic constraints to test the hypothesis above. Our assumptions are as follows. First, we assume a cellular objective of growth rate maximization, (max*μ* in (2)). This is the same cellular objective as ME in a static environment and DynamicME without inertia. Second, we assume that under exponential growth on various carbon sources, active protein degradation is negligible compared to dilution. Therefore, in (2) we have a decrease in protein abundance (*δ*_*i*_<0) only when dilution rate exceeds complex formation rate, i.e., when $\mu p_{i} > v_{i}^{\text {form}}$.

Based on these assumptions, we investigated how the proteome dynamics constraints, referred to as protein “inertia”, altered dynamic cellular responses to environmental fluctuations.

### Protein inertia changes the optimal proteome allocation

The first change due to protein inertia was an overall dampening of protein expression responses, as expected by the additional dependency of protein concentrations on those of the previous time step (2). We also observed two more important effects of inertia constraints: altered proteome allocation and metabolic mode.

**Fig. 7:**
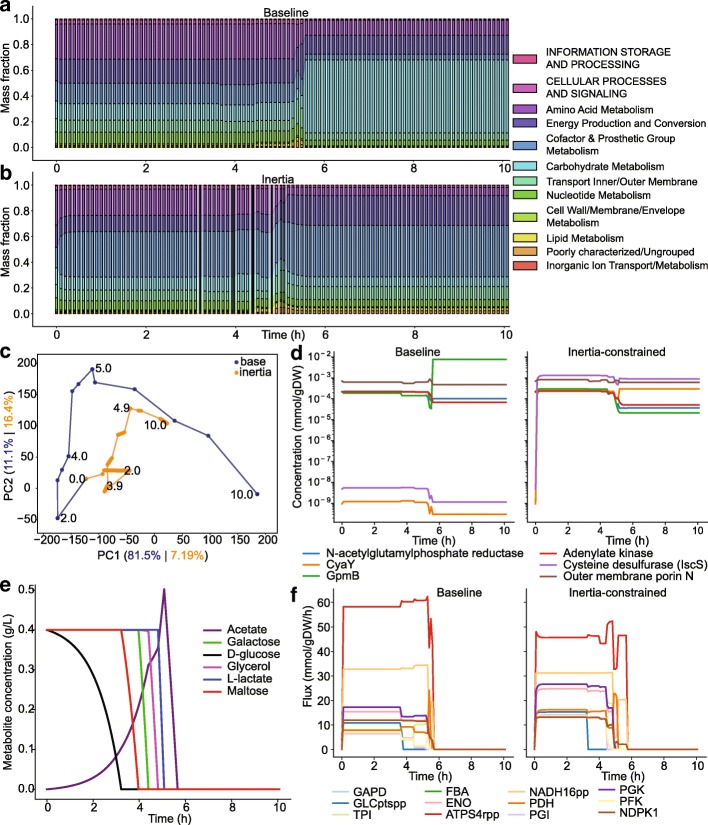
DynamicME simulations with protein dynamics constraints. Mass fraction of protein groups (by metabolic subsystem as in [49]) for baseline (**a**) and inertia-constrained models (**b**). (**c**) Principal component analysis (PCA) of protein concentrations. Percent variance explained is shown in the axis labels. These values were computed using principal components computed from the baseline data. Time points (in hours) are shown next to the markers. (**d**) Select protein concentrations that differed markedly between models. (**e**) Extracellular metabolite concentrations simulated by the inertia-constrained model. (**f**) Select metabolic fluxes that differed markedly between models. GAPD: glyceraldehyde-3-phosphate dehydrogenase. GLCptspp: glucose transport by phosphotransferase system. TPI: triose-phosphate isomerase. FBA: fructose-biphosphate aldolase. ENO: enolase. ATPS4rpp: ATP synthase. NADH16pp: NADH dehydrogenase (ubiquinone). PDH: pyruvate dehydrogenase. PGI: glucose-6-phosphate isomerase. PGK: phosphoglycerate kinase. PFK: phosphofructokinase. NDPK1: nucleoside-diphosphate kinase (ATP:GDP)

First, the proteome composition attained by the end of the batch was itself different, as evidenced by the allocation of protein groups involved in metabolic and expression processes (Fig. 7a-b). Principal components analysis (PCA) also confirmed that while major shifts in proteome allocation occurred at similar time points for both models, their overall directions of change differed considerably (Fig. 7c). A closer examination showed that a major effect of protein inertia constraints was higher investment in cofactor and prosthetic group synthesis very early in the batch culture (Fig. 7b). Specifically, inertia constraints led to higher synthesis of cysteine desulfurase (IscS) and the CyaY protein, which transfer sulfur and Fe(II) groups during iron-sulfur cluster biosynthesis, respectively.

Second, and related to this protein expression change was a notable shift in metabolic mode. Inertia-constraints led to lowered oxidative phosphorylation (lower ATP synthase, and NADH dehydrogenase fluxes) and higher substrate-level phosphorylation as evidenced by increased fluxes through phosphoglycerate kinase, phosphofructokinase, and overall higher flux through glycolysis (Fig. 7f). As a result, acetate accumulation was considerably higher than without inertia constraints (Fig. 7e), almost matching the extracellular acetate concentrations observed in experiments by Beg et al. [9].

The predicted alteration in metabolic mode was consistent with the altered proteome allocation. Specifically, pyruvate dehydrgenase (PDH) flux was predicted to increase under inertia constraints (on average 1.8-fold higher than without inertia constraints over all timepoints) (Fig. 7f). The PDH complex consists of three protein subunits (E1, E2, and E3), each having multiple copies [50]. In particular, lipoate moieties are attached to the E2 (AceF) subunit. In turn, lipoate synthesis is catalyzed by lipoyl synthase (LipA), which requires an iron-sulfur cluster. Thus, the increased requirement for lipoate synthase enzymes explains elevated levels of iron-sulfur cluster synthesis proteins IscS and CyaY.

## Discussion

In this study we developed DynamicME, an algorithm for simulating time-course metabolic and proteomic profiles using genome-scale models of metabolism and macromolecular expression (ME-models). We found that DynamicME correctly predicted substrate utilization hierarchy under a five-carbon mixed substrate medium. The biological basis for this hierarchy was proteome-limited cellular growth.

To account for the tendency of constraint-based models, including ME-models, to over-predict metabolic efficiency over wild-type cells, as well as parameter uncertainty, we implemented a model calibration procedure. In this study we focused on perturbing the effective rate constants (*k*_eff_) to match metabolite concentration profiles better. We arrived at a set of models showing improved prediction of the substrate utilization hierarchy. We note that sensitivity of ME model predictions to *k*_eff_ values has been investigated in several studies including a non-dynamic context [44], in relation to expression of protein groups (or sectors) [49], and for defining a core proteome [45]. However, the sensitivity of the predicted sequence and preference of utilizing mixed carbon substrates over time had not been investigated prior to the present study.

A notable feature of DynamicME is its ability to predict time-course proteome allocation profiles. We observed good agreement between measured and computed time-course expression profiles (median lagged cross-correlation of 0.67). Meanwhile, one subtle difference between measured and predicted time-course profiles was that measured profiles changed less abruptly due to process time constants of transcription and translation dynamics.

Finally, we investigated how proteome dynamic (“inertia”) constraints affect the capacity of *E. coli* to dynamically adjust its proteome allocation, and how this affects metabolism. At first glance, it is not intuitive how the protein inertia constraints (2) would alter the predicted metabolic and proteomic states, other than perhaps a simple smoothing operation of the protein abundances over time. However, because the optimization problem at each time step now accounts for a limited change in protein re-allocation at a future time point, the optimal solution will be quite different from that when the proteome can reallocate freely. Furthermore, because we do not allow active protein degradation by proteases, the only way to decrease protein abundance is by diluting the protein (at a rate exceeding translation), which further limits change of protein abundance. Overall, protein inertia led to higher reliance on substrate-level phosphorylation and reduced oxidative phosphorylation. Coupled to this altered metabolic response was higher requirements for cofactor and prosthetic group biosynthesis, and higher secretion of acetate as a by-product. These altered responses more closely resembled experimental measurements compared with the baseline model. This result reinforces previous studies [48, 51] showing that dynamic protein expression constraints represent a biological phenomenon that is potentially important for determining dynamic cellular states under changing environments.

### Computational challenges

One of the challenges for DynamicME, and indeed ME models in general, is the large computational cost compared with metabolic models that do not account for the protein expression network. Without protein inertia constraints, the batch simulation (batch time of 10 h) required approximately 40 min with a simulation time step of 0.1 h. With protein inertia constraints, this batch simulation required 7 h. This increased computational cost for the inertia-constrained model stems from solving the ME model at every time step (i.e., for 100 time steps).

The primary reason for the large computational cost of ME models is that they are ill-conditioned [52]. The reason for ill-conditioning is the wide range in magnitude of coefficients in the stoichiometric matrix. As a result, decision variables take on values ranging 15 orders of magnitude or more [22]. For this reason, ME models are typically solved using quad-precision optimization solvers [25]. Quad-precision solvers require more computational effort than their double-precision counterparts. Some methods have shown that double precision solvers can solve ME models with around 10^−6^ infeasibility, but this infeasibility tolerance is usually too large for ME models—hence the use of quad-precision. Additionally, ME models include nonlinear constraints as functions of the growth rate but because they are quasi-convex constraints, the models are computed efficiently using bisection [22–24] or augmented Lagrangian methods [22]. Nonetheless, additional solver iterations are required at each time step of DynamicME in order to solve the ME model. These two computational costs are magnified by the larger size of the ME model networks.

### Macromolecular expression models and the extension to dynamic allocation

A number of frameworks exist that model cell metabolism and macromolecular allocation at the genome-scale. In addition to the ME framework [2] that provides the reconstructed metabolic and protein expression network for this work, a large number of studies have examined metabolism and macromolecular resource allocation. Please see ref. [13] for a more comprehensive review of such modeling frameworks in non-dynamic contexts, as the scope of this work focuses on the dynamic extension of such models.

A representative method for integrating macromolecule allocation with metabolism (distinct from ME) is resource balance analysis (RBA). RBA extends flux balance analysis [53] with additional constraints and reactions to account for macromolecule synthesis and allocation [54]. A genome-scale RBA model of *Bacillus subtilis* included 614 reactions and 672 protein-coding genes and modeled cellular processes of metabolism and macromolecular processes (translation, protein folding, ribosome maturation, etc.) [23].

RBA has been used for a number of studies, including estimating in vivo apparent catalytic rates for *B. subtilis* by integrating proteomics and fluxomics, predicting the hierarchy of using carbon and nitrogen sources, predicting switches between metabolic pathways at the genome-scale, among others [55]. RBA was also used to examine the hierarchy of utilizing multiple carbon sources by integrating combinatorial optimization concepts based on a Boolean formalism [24].

Very recently, a dynamic modeling framework was developed for RBA, called dynamic RBA (dRBA) [56]. The dRBA method was demonstrated on a simplified model of a cell consisting of four fluxes representing conversion of a single substrate into macro-components and a product of interest [56].

In this context, the advancements of our paper are: (i) implemented the dynamic simulation of metabolism and macromolecular expression on a genome-scale ME model having up to 12,677 metabolic and protein expression reactions, (ii) showed that protein inertia (i.e., limitation in change of protein abundance over time due to capped synthesis and dilution rates) can cause a shift in the metabolic mode (lower oxidative phosphorylation, higher acetate overflow), and (iii) we provide the software (publicly on Github) with the aim of broader adoption by the community (see Availability of data and materials).

### Future directions

Overall, there is continuing need to develop efficient computational methods for algorithms that utilize ME models, such as dynamic simulations. A number of studies have developed methods for dynamic simulation of integrated models of metabolism and protein expression [20, 23, 24]. Time-scale decomposition and collocation approaches have a rich history in the dynamic modeling domain, and they have been applied to metabolic and expression networks [21]. We hope that future studies will continue to extend such methods for increasingly larger, integrated models of metabolism and protein expression. In particular, the addition of protein inertia constraints significantly increased the computational cost, and this framework may be improved in future studies.

Besides computational methods, DynamicME may be extended further to account for active protein degradation [57] and dynamic stress responses [58]. For example, under conditions of starvation stress starvation [59] or thermal stress [57], active protein degradation becomes important. Furthermore, during the transition between multiple carbon sources, *E. coli* was shown to up-regulate generic stress response genes [9]. Such extensions would allow the framework to be applied to non-growth phenotypes, where active stress responses including protein homeostasis become important for reallocating the proteome to perform cellular tasks besides biomass synthesis [59].

## Conclusions

ME-models compute cellular resource allocation tradeoffs at the proteome scale [13]. This expanded biological scope and predictive capability of ME models is expected to become increasingly useful for biotechnological applications [60]. For example, the metabolic and proteomic burden to the host of expressing biochemical production pathways can be computed explicitly using ME-models. We have shown here that it is furthermore possible to compute how these genome-wide cellular resource dynamics determine transient shifts in metabolic modes under biotechnologically relevant culture conditions: complex media with transient substrate availability. Thus, as ME-models continue to be reconstructed for organisms of biotechnological importance, DynamicME will be a useful approach for analyzing physiological and omics data from cell culture, and for model-aided biotechnological applications that require robust cell factory operation under environmental fluctuations [61]. DynamicME may also be useful for studying protein expression dynamics that are relevant for infectious disease (e.g., persister states [59]), especially by extending the protein inertia procedure to account for active protein degradation, or by utilizing a ME model that includes stress response mechanisms [57, 58].

## Additional files

Additional file 1 **Table S1.** Reactions with perturbed *k*_eff_. A subset of the genome-scale metabolic network was perturbed with respect to *k*_eff_ values, either manually or randomly. (XLSX 17 kb)

Additional file 2 **Figure S1.** Scree plot for determining number of archetypes. A notable elbow is observed for five archetypes. (PDF 5 kb)

Additional file 3 **Figure S2.** Parameter estimation procedure. We developed a parallel implementation of a metaheuristic optimization procedure. L-TA: list-based threshold accepting algorithm [37]. Variable definitions: *T*^*k*^, threshold value at iteration *k*; *T*^max^, maximum threshold value; *Z*^0^, objective value at current solution; *Z*^*k*^, objective value at neighboring solution (generated from current solution) at iteration *k*. (PDF 189 kb)

Additional file 4 **Figure S3.** Parameter estimation results. The parallel L-TA optimization procedure successfully estimated model parameters that improved consistency with measured concentration profiles. Seven parallel nodes were used here: 1 local and 6 global nodes (see Additional file 3: Figure S2) for explanation of nodes. (PDF 124 kb)

Additional file 5 **Table S2.** Lagged cross-correlation values. Cross-correlation values for fixed lag time of 1.2 h. (XLSX 41 kb)
