# Supplementary figures and images for: DynamicME: dynamic simulation and refinement of integrated models of metabolism and protein expression
Source: BMC Syst Biol. 2019 Jan 9;13:2. doi: 10.1186/s12918-018-0675-6 (PMC6327497; doi:10.1186/s12918-018-0675-6)

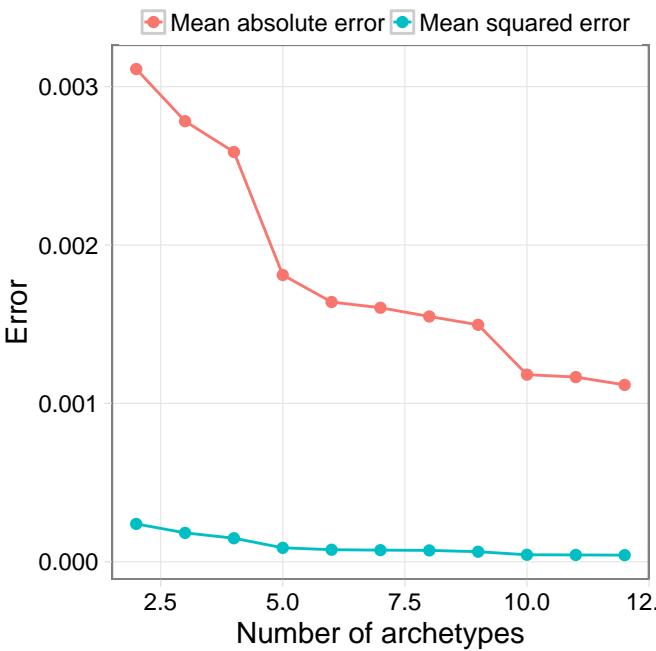

Supplement: Supplementary file 2 — Figure S1. Scree plot for determining number of archetypes. A notable elbow is observed for five archetypes. (PDF 5 kb) [file 12918_2018_675_MOESM2_ESM.pdf]

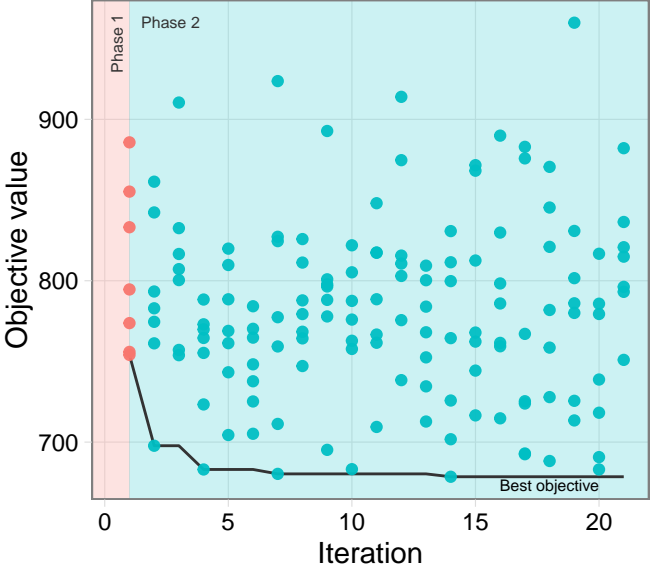

Supplement: Supplementary file 4 — Figure S3. Parameter estimation results. The parallel L-TA optimization procedure successfully estimated model parameters that improved consistency with measured concentration profiles. Seven parallel nodes were used here: 1 local and 6 global nodes (see Additional file 3: Figure S2) for explanation of nodes. (PDF 124 kb) [file 12918_2018_675_MOESM4_ESM.pdf]
