# Supplementary material for: DynamicME: dynamic simulation and refinement of integrated models of metabolism and protein expression
Source: BMC Syst Biol. 2019 Jan 9;13:2. doi: 10.1186/s12918-018-0675-6 (PMC6327497; doi:10.1186/s12918-018-0675-6)

L-TA procedure

1. Perturb parameters
2. Compute new solution
3. Compute prediction error
4. $T^k = \frac{z^k - z^0}{z^0}$.

   IF $T^k \leq T^{max}$:
   - Accept move.
   - If $T^k > 0$: append T^k to list and remove T^{max}.

   ELSE:
   - Reject move (return to previous point).

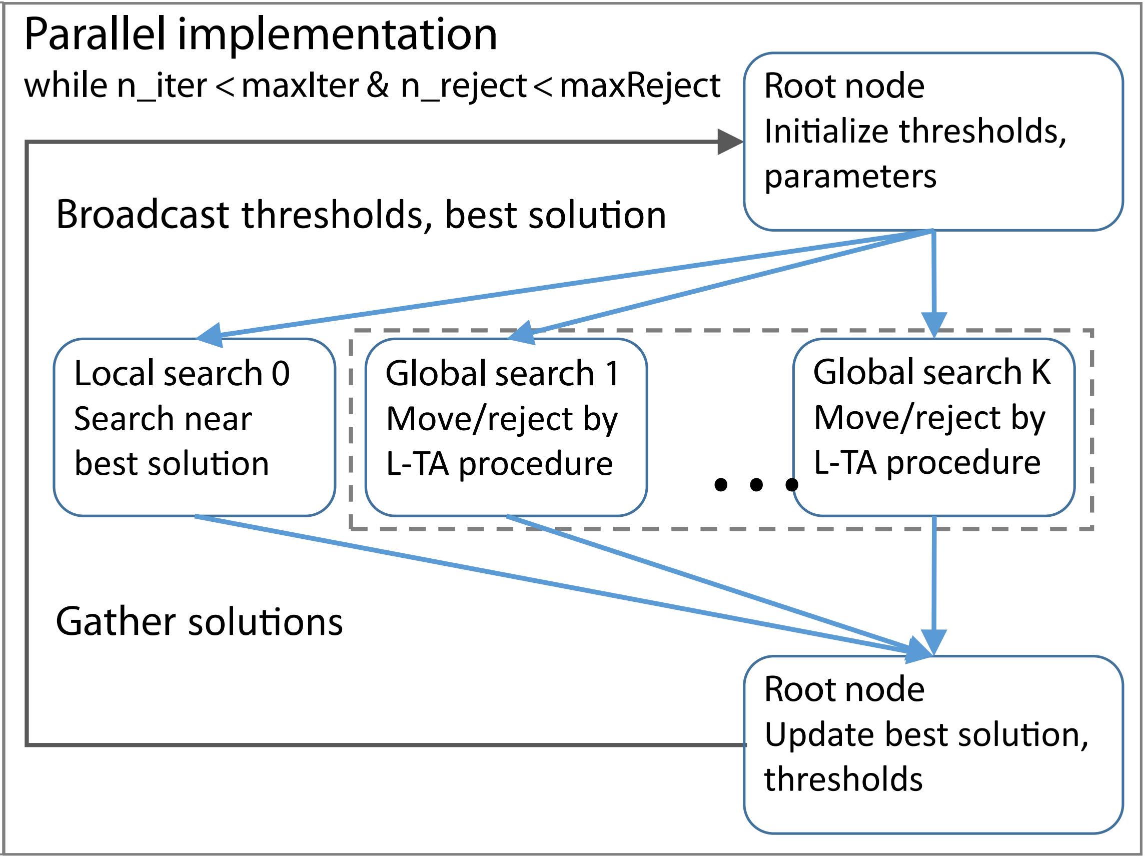

Supplement: Supplementary file 3 — Figure S2. Parameter estimation procedure. We developed a parallel implementation of a metaheuristic optimization procedure. L-TA: list-based threshold accepting algorithm [37]. Variable definitions: Tk, threshold value at iteration k; Tmax, maximum threshold value; Z0, objective value at current solution; Zk, objective value at neighboring solution (generated from current solution) at iteration k. (PDF 189 kb) [file 12918_2018_675_MOESM3_ESM.pdf]
